# Navigating the path towards successful implementation of the EU HTA Regulation: key takeaways from the 2023 Spring Convention of the European Access Academy

**DOI:** 10.1186/s12961-024-01154-2

**Published:** 2024-07-02

**Authors:** Francine Brinkhuis, Elaine Julian, Hendrika van den Ham, Fabrizio Gianfrate, Valentina Strammiello, Michael Berntgen, Mira Pavlovic, Peter Mol, Jürgen Wasem, Walter Van Dyck, Antonella Cardone, Christian Dierks, Anja Schiel, Renato Bernardini, Oriol Solà-Morales, Jörg Ruof, Wim Goettsch

**Affiliations:** 1 Utrecht WHO Collaborating Centre for Pharmaceutical Policy and Regulation, Division of Pharmacoepidemiology and Clinical Pharmacology, Utrecht University, Utrecht, The Netherlands; 2 Secretariat of the European Access Academy (EAA), Hauensteinstr. 132, 4059 Basel, Switzerland; 3 University of Ferrara, Ferrara, Italy; 4 European Patients’ Forum (EPF), Brussels, Belgium; 5 European Medicines Agency (EMA), Amsterdam, The Netherlands; 6 Medicines Development and Training (MDT) Services, Paris, France; 7 Department of Clinical Pharmacy and Pharmacology, University Medical Center Groningen (UMCG), University of Groningen, Groningen, The Netherlands; 8 Institute for Health Care Management and Research, University of Duisburg-Essen, Essen, Germany; 9 Healthcare Management Centre, Vlerick Business School, Brussels, Belgium; 10 Cancer Patients Europe’ (CPE), Brussels, Belgium; 11 Dierks + Company, Berlin, Germany; 12 Norwegian Medicines Agency (NOMA), Oslo, Norway; 13 Department of Biomedical and Biotechnological Sciences (BIOMETEC), Section of Pharmacology, University of Catania, Catania, Italy; 14 HiTT Foundation, International University of Catalonia-UIC, Barcelona, Spain; 15 Medical School of Hanover, Hanover, Germany; 16 National Health Care Institute, Diemen, The Netherlands

**Keywords:** EUHTA, Health Policy, Stakeholder involvement, Health Technology Assessment, Access

## Abstract

**Background:**

The European Regulation on Health Technology Assessment (EU HTA R), effective since January 2022, aims to harmonize and improve the efficiency of common HTA across Member States (MS), with a phased implementation from January 2025. At “midterms” of the preparation phase for the implementation of the Regulation our aim was to identify and prioritize tangible action points to move forward.

**Methods:**

During the 2023 Spring Convention of the European Access Academy (EAA), participants from different nationalities and stakeholder backgrounds discussed readiness and remaining challenges for the Regulation’s implementation and identified and prioritized action points. For this purpose, participants were assigned to four working groups: (i) Health Policy Challenges, (ii) Stakeholder Readiness, (iii) Approach to Uncertainty and (iv) Challenges regarding Methodology. Top four action points for each working group were identified and subsequently ranked by all participants during the final plenary session.

**Results:**

Overall “readiness” for the Regulation was perceived as neutral. Prioritized action points included the following: Health Policy, i.e. assess adjustability of MS laws and health policy processes; Stakeholders, i.e. capacity building; Uncertainty, i.e. implement HTA guidelines as living documents; Methodology, i.e. clarify the Population, Intervention, Comparator(s), Outcomes (PICO) identification process.

**Conclusions:**

At “midterms” of the preparation phase, the focus for the months to come is on executing the tangible action points identified at EAA’s Spring Convention. All action points centre around three overarching themes: harmonization and standardization, capacity building and collaboration, uncertainty management and robust data. These themes will ultimately determine the success of the EU HTA R in the long run.

## Background

In December 2021, the European Parliament and the Council of the European Union (EU) adopted the European Regulation on Health Technology Assessment (EU HTA R (EU)2021/2282), which came into effect in January 2022 [1]. The EU HTA Regulation aims at promoting collaboration and harmonizing HTA practices across the EU by reconciling divergent national HTA approaches and establishing standardized methodologies and processes. Ultimately, the joint work requested by the Regulation aims to enhance effective use of resources and foster strong cooperation in HTA across the EU [2–4]. While the harmonized procedure covers the clinical aspects of HTA, i.e. relative clinical effectiveness and safety of a new health technology, Member States (MS) will remain responsible for conclusions on the overall added value (i.e. appraisals) and related decisions on pricing and reimbursement [2].

In January 2022, a 3-year transition period, referred to as the “preparation phase”, for the joint EU HTA process was initiated. It involves several critical activities to support the successful implementation of the EU HTA Regulation, including (i) the establishment of the Coordination Group consisting of MS’ representatives with its subgroups on Joint Clinical Assessments (JCAs), Joint Scientific Consultations (JSCs) and Identification of Emerging Health Technologies and Methodology, (ii) the formation of the HTA Stakeholder Network, (iii) the drafting of the Implementation and Delegated Acts, and (iv) the creation of Guidance Documents as outlined in the Implementation Rolling Plan [2, 4–8]. Starting from January 2025, with the preparation phase completed, JCAs will be carried out for all cancer medicines and advanced therapy medicinal products (ATMPs), followed by orphan drugs from January 2028 and, finally, all other centrally approved medicines and a selection of medical devices from 2030 onwards (Article 7.2) [2].

In September 2021, to support the implementation of the Regulation, the European network for Health Technology Assessment (EUnetHTA) 21 joint consortium was instituted, consisting of 13 European HTA agencies and led by the Zorginstituut from the Netherlands [4, 9, 10]. The work agenda of EUnetHTA 21 included several deliverables, including the development of methodological guidance and the conduct of a limited number of JCAs and JSCs [4, 11, 12]. Approximately at the same time, the European Access Academy (EAA) was established as a multi-stakeholder initiative with the primary objective of developing a joint European value framework to facilitate the assessment of innovative health technologies, thereby supporting the vision of the EU HTA Regulation to increase patient access to innovative and life-saving technologies [1, 4, 5]. The EAA held its inaugural convention in May 2022 followed by a second convention in October 2022. These events brought together experts from a range of stakeholder groups to accomplish two respective objectives: first, to develop a research agenda aimed at addressing key challenges in implementing the Regulation and the evolving HTA value framework, and second, to gather insights and develop a call to action for optimal stakeholder involvement [5–7, 13].

As the preparation phase towards the joint EU process approached its half-way mark in mid-2023, the focus of the third EAA Convention in April 2023, titled “Midterms & Status of the Preparation Phase of the EU HTA Regulation”, was on evaluating the progress already made and identifying steps and actions needed to support a successful implementation of the EU HTA regulation [13]. We present here the input received from the multi-stakeholder participants of the EAA Spring Convention 2023 Working Session on identifying and prioritizing key action points for the remaining months of the preparation phase.

## Methods

### Preparation of break-out sessions during the EAA Convention

The 2023 Spring Convention of the EAA was held on 21 April 2023 at Utrecht University (Utrecht, the Netherlands). The Convention included plenary sessions as well as break-out sessions with smaller working groups (WGs), both of which were designed as hybrid meetings to allow on-site and remote participation via Microsoft Teams. Four dedicated WGs with approximately 15–20 participants each were formed in advance, with the following focus topics: Health Policy Challenges (*WG 1, Health Policy*), Stakeholder Readiness (*WG 2, Stakeholders*), Approach to Uncertainty (*WG 3, Uncertainty*), and Challenges regarding Methodology (*WG 4, Methodology*). These themes were chosen on the basis of outcomes of previous EAA conventions and input from the EAA Faculty [4–7]. The goal of each break-out session was to identify and prioritize key challenges and corresponding action points relating to the EU HTA Regulation within its respective area. Allocation of participants to the four break-out sessions was based on the following criteria, with the aim to achieve equal distribution regarding each criterion among the working groups: (i) personal and professional background; (ii) national diversity in each group, (iii) stakeholder diversity within each group [patients and patients’ representatives, clinicians’ representatives, regulators, health technology developers (HTDs), HTA bodies, payers, policy makers, and academic representatives] and (iv) participation mode (i.e. on-site versus remote).

In preparation for the break-out sessions, two co-leads and a notetaker were appointed in advance for each group, taking into account national backgrounds, stakeholder group representation and professional expertise in the field to minimize potential bias. In pre-convention meetings between the EAA secretariat and the leadership teams, the proposed structure and approach of the break-out sessions were agreed upon to ensure a consistent approach and reporting across all sessions. Break-out co-leads were responsible for facilitating and structuring the sessions and for encouraging involvement of all attendees. Further, the notetaker was responsible for reporting key findings using a predefined PowerPoint format.

### Procedural approach of the break-out sessions

**Fig. 1:**
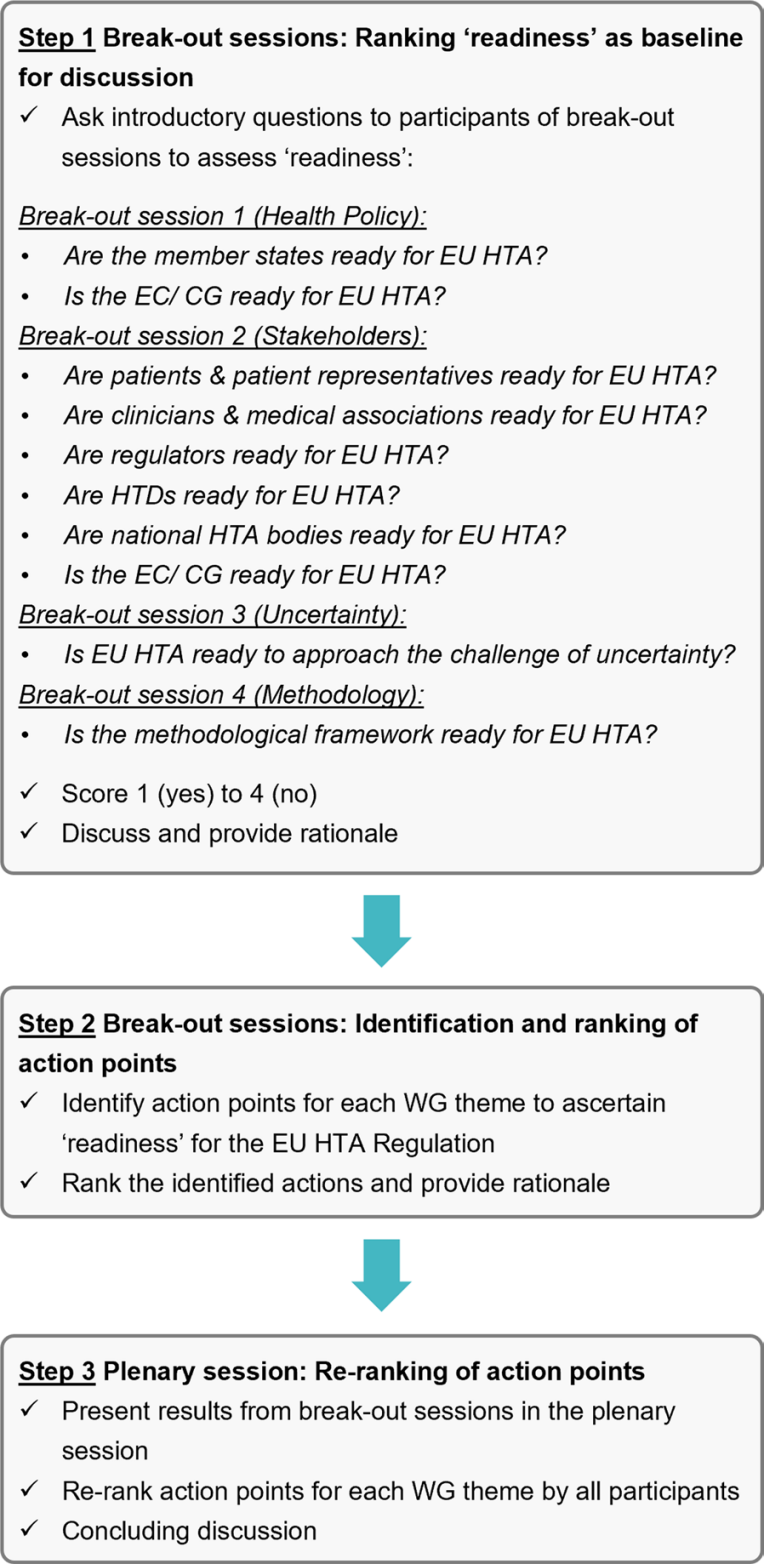
Three-step flow of the sessions that were held at the EAA Spring Convention 2023. CG, Coordination Group; EC, European Commission; EU, European Union; HTA, Health Technology Assessment; HTD, Health Technology Developer; WG, working group

The break-out sessions, scheduled for 120 min, aimed to facilitate meaningful discussions, encourage participant input, and generate actionable outcomes to be discussed at the plenary session. First, introductory ranking questions were posed using an IT-based system, in which participants were asked to rank the 'readiness' towards the Regulation within the area of their break-out session (step 1 of Fig. 1). The number of introductory ranking questions varied depending on the break-out session. The introductory questions were aimed to provide a consolidated overview of different participants’ perspectives on the topic of “readiness” in order to inform the subsequent discussions in the respective WG. Next, each WG developed a comprehensive list of action points addressing the remaining challenges within their area of focus (step 2 of Fig. 1). These action points were then prioritized, resulting in a top-four list. One representative was appointed for each WG to present the prioritized list in the final plenary session.

### Plenary session and ranking

Following the break-out sessions, the findings from the four respective WGs were reported back to the EAA plenary session by the representative of each group (step 3 of Fig. 1). Subsequently, the top four action points for each break-out session were formally re-ranked on the basis of their importance by all stakeholders present, both on-site and remote, using an IT-based system. The aggregated descriptive ranking data were visible to the audience in real time, fostering an informed and constructive discussion within the concluding plenary session during which key discussion points were reflected upon to further crystallize the proposed action points and identify any additional concerns.

### Data handling and analysis

The introductory questions to assess readiness towards the Regulation within the areas of the break-out sessions were conducted on an ordinal Likert response scale scoring from 1 (no) to 4 (yes). For the analysis, weights were allocated to each response corresponding to the value of the Likert scale and descriptive statistics including mean, median, max, min and upper and lower quartile were applied. The data for assigning the relative ranking scores during the plenary session were extracted from the online tool Slido [14]. Here, a weight was assigned to each item in proportion to its position in the ranking list as determined by each individual respondent, i.e. the highest item received the maximum points (4, as this was the number of pre-defined list items), while the lowest priority item was given a single point. Cumulative points for each option were then divided by the total number of respondents who participated in the ranking poll, thereby producing an average ranked score for each option. All ranking questions were presented via Slido and were shared via QR codes and HTML links on-site and remotely to allow for simultaneous IT-based ranking by all participants.

After the EAA Convention, the identified action points for each break-out session were extracted from the WG notes and were transferred to a table format. Any duplications were removed and several adjustments of wording were made to improve clarity. All responses received were pseudonymized prior to any analysis. Data were stored on a password-protected separate file. Data analysis was conducted by F.B. in consultation with E.J. and H.v.d.H.

## Results

### Break-out sessions

#### Attendance

**Fig. 2:**
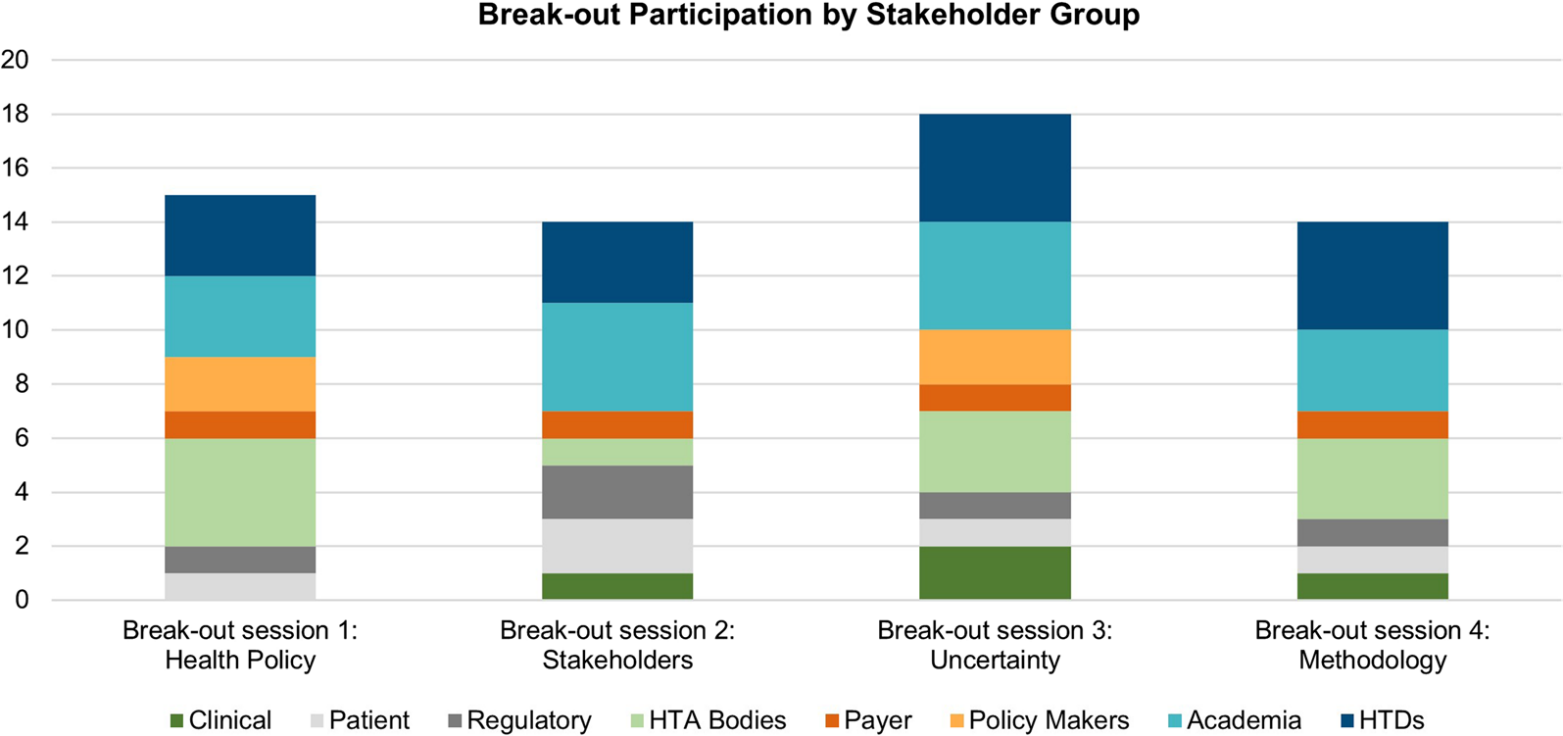
Representation of stakeholder groups in each break-out session. HTA, Health Technology Assessment; HTD, Health Technology Developer

The four break-out sessions at the EAA Spring Convention 2023 were attended by *N* = 15 (WG 1, Health Policy), *N* = 14 (WG 2, Stakeholders), *N* = 18 (WG 3, Uncertainty), and *N* = 14 (WG 4, Methodology) on-site and remote participants covering *N* = 14 national backgrounds (Belgium, Bulgaria, Denmark, France, Germany, Great Britain, Italy, Ireland, Malta, the Netherlands, Norway, Portugal, Spain, Switzerland). The distribution of representatives of each stakeholder group among the four break-out sessions is shown in Fig. 2.

#### Readiness towards the implementation of the EU HTA Regulation

**Fig. 3:**
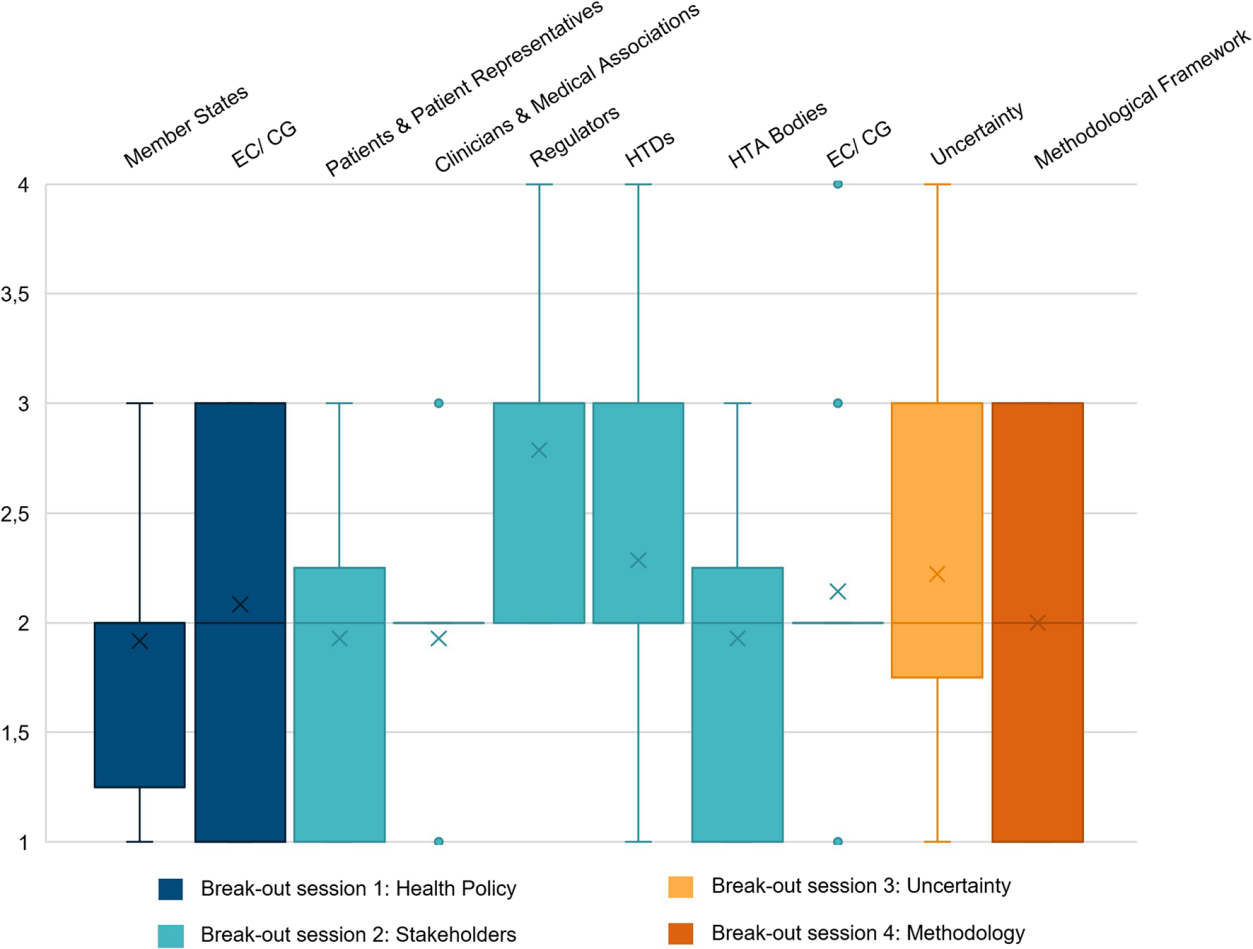
Box plots [mean (*x*); median; max; min; upper and lower quartile] for the ranking of the “readiness” within the areas of the four break-out sessions, relating to Health Policy (*N* = 12), Stakeholders (*N* = 14), Uncertainty (*N* = 18) and Methodology (*N* = 12), respectively. Rankings were conducted on an ordinal Likert response scale scoring from 1 (no) to 4 (yes). Note that the readiness of the EC/ CG was ranked in two distinct break-out sessions (see Fig. 1 for the questions per break-out session). CG, Coordination Group; EC, European Commission; HTA, Health Technology Assessment; HTD, Health Technology Developer

WG 1 (Health Policy) rated both the Readiness of Member States [MS; mean 1.9; interquartile range (IQR) 1.25–2; *N* = 12] and of the European Commission (EC)/Coordination Group (CG; mean 2.1; IQR 1–3; *N* = 12) rather low to neutral, although the latter showed higher variance. In WG 2 (Stakeholders), regulators as well as HTDs were perceived to have rather high readiness (mean 2.8; IQR 2–3; *N* = 14, and mean 2.3; IQR 2–3; *N* = 14, respectively). Patients and patient representatives were ranked low to neutral (mean 1.9; IQR 1–2.25; *N* = 14), as well as HTA bodies (mean 1.9; IQR 1–2.25; *N* = 14). The readiness of clinicians and medical associations (mean 1.9; IQR 2–2; *N* = 14) and of the EC/CG (mean 2.1; IQR 2–2; *N* = 14) was also perceived to be low to neutral, with very low variance in the responses obtained. In WG 3 (Uncertainty) and WG 4 (Methodology**)** the readiness of EU HTA to approach uncertainty (mean 2.2, IQR 1.75–3; *N* = 18) and the readiness of the methodological framework (mean 2, IQR 1–3; *N* = 12) were ranked low to neutral, with the latter exhibiting higher variance. Analysis of the ranking of readiness towards the implementation of the EU HTA Regulation is shown in Fig. 3.

#### Action points defined

The action points that were identified by each of the WGs are presented in Table 1. The top four action points as prioritized by each WG referred to:

- WG 1, Health Policy: (i) immediate implementation, (ii) shared standards and rules to appoint subgroup representatives, (iii) common success and failure criteria for the joint HTA work, (iv) adjustability of MS laws and policy processes and their willingness to change these;
- WG 2, Stakeholders: (i) raised awareness, (ii) sufficient capacity, (iii) alignment of national policies with EU-level, (iv) efficient communication by Coordination Subgroups;
- WG 3, Uncertainty: (i) living HTA guidelines, (ii) guidelines for real-world data (RWD), (iii) capacity/capabilities of physicians and HTDs, (iv) sufficient capacity for JSCs;
- WG 4, Methodology: (i) clarified Population, Intervention, Comparator(s), Outcomes (PICO) process, (ii) fit-for-purpose guidelines, (iii) link between JSCs, PICOs, and JCA preparations, (iv) usage of guidelines for addressing evidence gaps.

**Table 1:** Action points that were identified and prioritized during the four break-out sessions, relating to Health Policy (*N* = 15), Stakeholders (*N* = 14), Uncertainty (*N* = 18) and Methodology (*N* = 14)

Break-out session 1: Health Policy (*N* = 15)	Break-out session 2: Stakeholders (*N* = 14)	Break-out session 3: Uncertainty (*N* = 18)	Break-out session 4: Methodology (*N* = 14)
Prioritize – given the current phase of preparation – immediate implementation (i) Establish shared quality standards and rules to appoint subgroup representatives. The latter should be designated by MS (ii) Specify common success and failure criteria for the joint HTA work and require MS to report on performance (iii) Assess adjustability of MS laws and health policy and their willingness to change these (iv) Establish a survey process to evaluate the adoption of JSC and JCA advice in MS Foster an interactive collaborative process to politically drive convergence of MS HTA procedures towards a common EU HTA process Empower assessors to lead the convergence process, while ensuring they receive guidance on methodology application and consensus-building	*All stakeholder groups* Raise awareness among all stakeholder groups (i) Ensure sufficient capacity (training and resources) for all stakeholder groups (ii) *Patients* Improve capacity building of patient experts Manage conflicts of interest *Clinicians* Improve capacity building Develop scoring models (e.g. ESMO-MCBS) for other conditions *Regulators* Continue exchange with HTA bodies on the regulatory outcomes *HTDs* Engage in and provide comments on the new procedures through internal training and education Foster close interaction between regulatory affairs and market access departments Consolidate or establish new collaborations and networks at the EU and national level with HTA bodies and scientific societies *National HTA bodies* Work in English Establish clusters of HTA bodies Take action to revise national policies to ensure alignment with the EU level (iii) *EC/CG* Ensure sufficient capacity and funding Share information on progress Ensure efficient communication of progress by the Coordination Subgroups (iv) Identify incentives for HTA bodies to use the JSC and JCA advice	Implement HTA GLs as living documents in collaboration with stakeholders (i) Develop GLs for deriving evidence from RWD and managing associated uncertainty (ii) Enhance capacity/capabilities of physicians and HTDs to increase data robustness. Here, focus should also be on improving patient participation (iii) Establish sufficient capacities for JSCs to address individual cases (iv) Establish criteria for acceptable levels of uncertainty	Clarify the PICO identification process and its impact on JCAs (i) Evaluate the fitness-for-purpose of all guidelines, with emphasis on ATMPs and orphan drugs (ii) Establish a link between JSCs, potential PICOs, and JCA preparations. This should include PLEG requirements and focus on a lifecycle approach (iii) Define the use of guidelines in addressing evidence gaps. Ideally prior to initiating JCAs, including additional requirements beyond trial evidence (iv) Collect input of assessors (those not involved in the drafting) on the GLs Involve patients and patient representatives in the development of methodology guidelines Address challenges specific to ATMPs/orphans, requiring up-to-date, agile and flexible processes Ensure JSC teams have familiarized themselves with the GLs Plan for the incorporation of additional evidence when it becomes available

The positions of the action points on respective working groups’ priority lists are indicated with Roman numerals (i–iv). ATMPs, advanced therapy medicinal products; ESMO-MCBS, European Society for Medical Oncology-Magnitude of Clinical Benefit Scale; EU, European Union; GL, guideline; HTA, Health Technology Assessment; HTD, Health Technology Developer; JCA, Joint Clinical Assessment; JSC, Joint Scientific Consultation; MS, Member States; PICO, Population, Intervention, Comparator(s), Outcomes; PLEG, Post Launch Evidence Generation; RWD, real-world data

### Plenary session

#### Ranking of action points

**Fig. 4:**
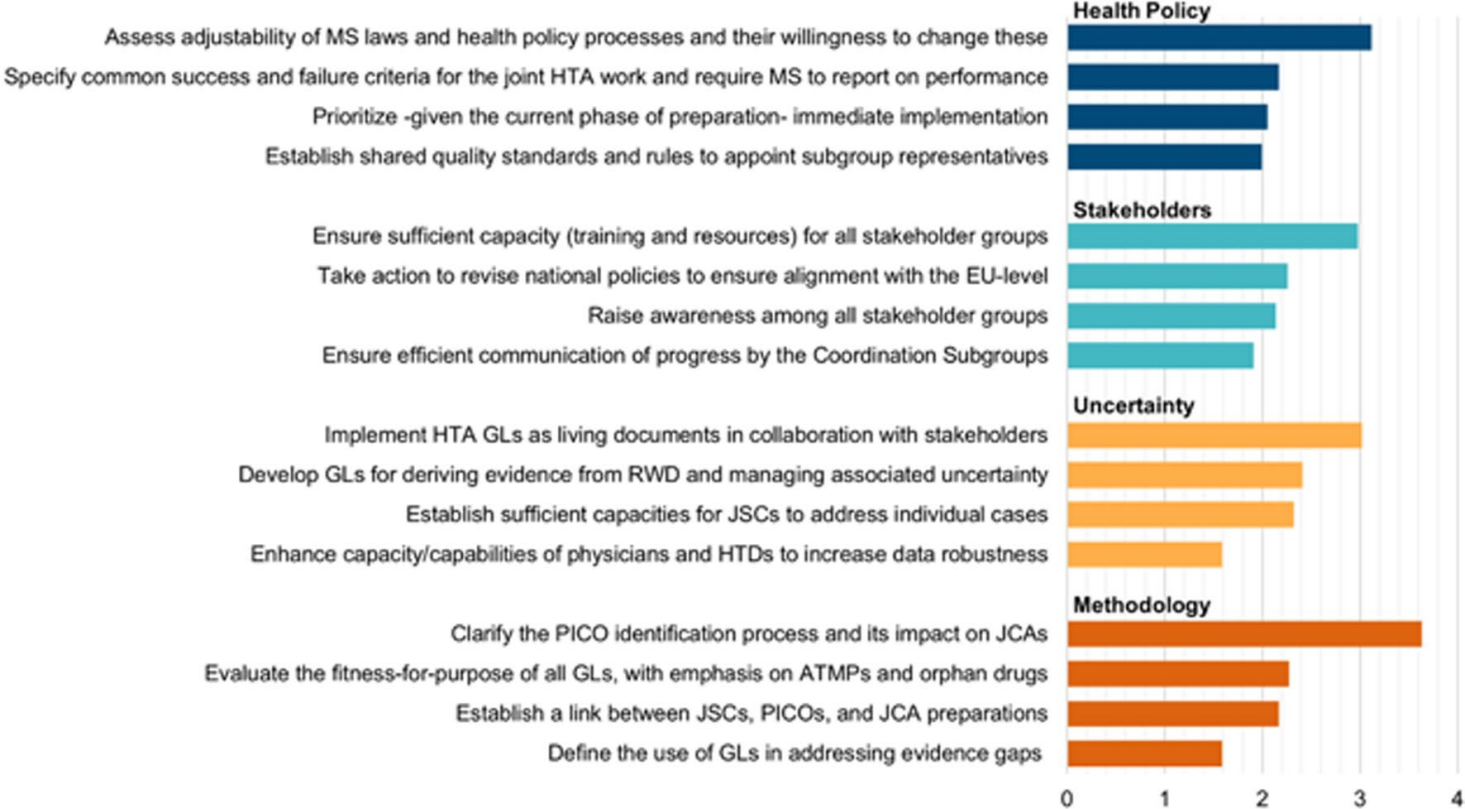
Relative importance of key action points within the area of focus for each break-out session, as prioritized by the respondents during the plenary session. Cumulative weighted responses for each list item were divided by the total votes (Health Policy:* N* = 40; Stakeholders:* N* = 43; Uncertainty:* N* = 41; Methodology:* N* = 41) to express an average of the maximum possible weight, i.e., 4 points. ATMPs, Advanced Therapy Medicinal Products; EU, European Union; GL, Guideline; HTA, Health Technology Assessment; HTD, Health Technology Developer; JCA, Joint Clinical Assessment; JSC, Joint Scientific Consultation; MS, Member States; PICO, Population, Intervention, Comparator(s), Outcomes; PLEG, Post Launch Evidence Generation; RWD, Real-World Data

The top four action points as re-ranked in the plenary session are shown in Fig. 4. The top prioritized action point for each WG are:

- WG 1, Health Policy: assess adjustability of MS laws and health policy processes and their willingness to change these (score 3.1).
  - Participants stressed the importance of assessing the preparedness of MS to embrace future aligned policies. Readiness of MS for the Regulation was considered to depend mostly on the degree to which national decision-making processes and legal frameworks need to be adapted by 2025.
- WG 2, Stakeholders: ensure sufficient capacity (training and resources) for all stakeholder groups (score 3.0);
  - Participants emphasized that various stakeholder groups face limitations in terms of capacity, including resources, expertise and manpower, which hinder their participation in the collaborative efforts envisioned by the Regulation.
- WG 3, Uncertainty: implement HTA guidelines as living documents in collaboration with stakeholders (score 3.0);
  - In addition to ensuring that guidelines are fit-for-purpose, it was emphasized that these should be living documents. This will enable the continuous incorporation of emerging best clinical practices and guarantee that the guidelines remain relevant and up to date.
- WG 4, Methodology: clarify the PICO identification process and its impact on JCAs (score 3.6).
  - Participants highlighted that it is currently unclear whether all PICOs that are requested by MS will be included in the assessment. Should not all PICO schemes be included, it was considered unclear how the divergent needs of MS, including varying subgroups or comparators, would be prioritized, and whether this prioritization process would be conducted in a fair and transparent manner.

## Discussion

By consolidating the strengths of national HTA processes into a harmonized EU-wide common HTA approach, the Regulation aims to minimize duplication of efforts by HTA agencies and HTDs, enhance predictability, promote the long-term sustainability of EU-wide HTA cooperation and, ultimately, facilitate equal access to innovative health technologies within the EU [3, 15]. For this goal to be achieved successfully, it is crucial to define tangible strategies to tackle the key challenges that remain, bearing in mind the advanced stage of the preparation phase. The data presented in this article underline prioritized action points as identified by participants from the EAA Spring Convention 2023, representing diverse stakeholder groups that are closely associated with the Regulation. Noteworthy findings especially underscore the need to assess adaptability of MS laws and policy processes and willingness to change these, sufficient capacities of stakeholders in terms of training and resources, the adoption of living guidelines co-created by stakeholders, and clarification of the PICO scoping process and its impact on JCAs.

### Readiness towards EU HTA

Our findings indicate a prevailing neutrality in readiness for the EU HTA Regulation, with mean scores ranging between 1.9 and 2.3 for almost all introductory questions, except one. Notably, the readiness of Regulators received the highest rating of readiness with a mean score 2.8, given their extensive preparations for the Regulation. Indeed, the European Medicines Agency and EUnetHTA 21 have been engaged in collaborative efforts since 2010, involving the exchange of information and discussions on matters of mutual interest and aiming to align regulatory evaluations and HTA assessments, working towards harmonization in these domains [16–18]. It is, however, important to note that Regulators are not included in Preamble §44 of the EU HTA Regulation and would therefore technically be considered collaborators/contributors rather than stakeholders [2, 5, 7]. Nonetheless, fostering strong collaboration between Regulators and HTA bodies is considered a key success factor for the Regulation, as continuous involvement in the regulatory process plays a crucial role in facilitating an efficient and predictable JCA process for all stakeholders concerned [4–6, 19–25]. The JCA timelines, as currently proposed, are indeed based on and closely linked to the regulatory timelines in pursuit of the aforementioned objectives [25].

### Identification/prioritization of action points

During the break-out sessions of the EAA Convention, the respective WGs identified several key action points that centred around three overarching themes: (i) harmonization and standardization, (ii) capacity building and collaboration, and (iii) uncertainty management and robust data.

#### Harmonization and standardization

Promoting convergence of HTA tools, procedures and methodologies stands as a critical objective within the Regulation [23], addressing an urgent need due to the notable disparities that exist among HTA working practices and recommendations across different European jurisdictions, inevitably leading to inequities in patient access [6, 26–28]. Previous studies have extensively focused on understanding the variations in HTA practices across Europe to identify opportunities for further alignment [26, 29]. In the context of clinical HTA assessments, cross-country differences mainly stem from disparities in the evidence assessed, interpretation of evidence, methodological approaches of the assessment, uncertainty management, and the extent to which other considerations, such as disease severity, available treatments and unmet need, influence assessments [29–31]. These variations naturally arise from the unique political and social values of different jurisdictions, as well as specific needs within each healthcare context [26, 31–33]. While efforts have been made to develop methodological and procedural guidelines under EUnetHTA 21 to facilitate the implementation of the EU HTA Regulation, it is not yet clear to what extent these will be adopted by the EC in the Implementing Acts and by the CG in respective guidance documents and, therefore, to what extent these will align with the requirements of individual MS [2, 12]. Consequently, despite the Regulation’s objective to promote convergence of HTA procedures, it will likely be inevitable for some MS to “perform complementary clinical analyses relating, inter alia, to patient groups, comparators or health outcomes other than those included in the joint clinical assessment report, or using a different methodology if that methodology would be required in the overall national HTA process of the Member State concerned” as outlined in Preamble §15 of the Regulation [2].

One of the approaches to address the diverging needs of MS is through the PICO survey, aiming to identify each MS PICO requirements and subsequently consolidate them into the minimum number of PICOs necessary to meet the needs of as many MS as possible [34, 35]. Numerous concerns have emerged regarding the possibility of MS requesting multiple divergent PICO schemes, which is considered one of the major hurdles for successful implementation of the Regulation [4, 7, 36]. Indeed, a multiplicity of PICOs significantly increases the time and resources required for HTDs to collect, analyse and present the requested data when available, as well as the time and resources for HTA agencies to assess the data for each PICO, leading to a substantial workload which may result in increased complexity rather than a simplification of the HTA process [4, 7, 36]. The difficulties of aligning PICO elements have been the subject of extensive research and of discussions at previous EAA Conventions in 2022 [4, 5, 7, 20, 21, 36], which primarily focused on the level of agreement of PICO parameters between jurisdictions and the feasibility of managing multiple PICOs. Our study complements previous research by revealing that the scoping process of PICOs is not only considered challenging in terms of feasibility but is also viewed as unclear, creating hurdles in stakeholders’ readiness for the Regulation. For instance, HTA agencies may encounter challenges in predicting the extent of supplementary clinical analyses required for their national procedures as long as the prioritization process of divergent MS’ needs in terms of PICO parameters remains unclear. Therefore, we advocate the EC/CG to engage in clear and transparent communication regarding the PICO scoping process, thereby enhancing overall clarity for stakeholder groups and fostering their readiness for the Regulation.

#### Capacity building and collaboration

Participants highlighted the critical issue of capacity shortages among all stakeholder groups. This deficiency may, in turn, be closely related to a lack of awareness of some stakeholders concerning the EU HTA Regulation or even HTA in general, which was also identified as an action point. For example, clinicians and patients are anticipated to play vital roles within the new framework, with patient and clinical experts being given the opportunity to provide input on JCAs and JSCs, whereas patient organizations and medical societies can contribute insights through the Stakeholder Network [37]. However, patient involvement practices within the field of HTA are currently limited across different European jurisdictions due to a number of reasons, including financial/time constraints, lack of capacity, poor training/support, language barriers and a generally low awareness [5, 38–40], while similar factors impede the involvement of clinicians and medical organizations in HTA practices [5, 7]. Adding to the complexity, several technical and practical challenges of the involvement of both stakeholder groups were highlighted; for example, the definition and qualification criteria of a “patient expert” are vague, the involvement of clinicians and patients in the PICO process lacks clarity and may potentially lead to conflicts of interest – points of criticism that have also been voiced during the inaugural meeting of the Stakeholder Network as well as in prior studies [5, 7, 41]. On the whole, this hampers the involvement of patients/patient organizations and clinicians/medical societies within the domain of HTA and the Regulation, underscoring the need for not only well-structured training programmes and sufficient capacity, but also methodologies to guide the involvement of both stakeholder groups [7, 37].

Our results indicate that poor stakeholder involvement due to a lack of awareness and/or capacity is not only evident for patients and clinicians, but also for the other stakeholder groups. The pressing need to increase awareness of all stakeholders by improved training and capacity building has previously been highlighted as a critical action point by the Stakeholder Network and during the 2022 Fall Convention of the EAA [7, 41]. In its pursuit to “reach out to all relevant national authorities and stakeholders so that they may not only provide their input, but also become partners in this process” [42], the Commission acknowledges the vital role that stakeholders play in realizing the goals of the Regulation, highlighting the significance of their alignment within the overarching objectives of the EU HTA Regulation. A recent study by Hogervorst et al. (2023) highlighted the significance of institutionalized communication as a key element in enhancing synergy among stakeholders. The study further advocates for the early initiation of multi-stakeholder dialogues, along with the adoption of shared definitions and methods [24]. The Commission has already indicated that efforts are made to organize regional information sessions to inform and raise awareness about HTA among local stakeholder communities [41]. Given the reaffirmation of this action point as a high-priority concern during the present convention, coupled with the advanced stage of the preparation phase, we advocate for escalated efforts aimed at improving communication, raising awareness and elevating training/capacity building.

#### Uncertainty management and robust data

Uncertainty constitutes a fundamental element in HTA-informed decision-making, and while all HTA agencies share a common concern regarding uncertainty management, the strategies employed to decrease it and the levels of uncertainty that can be accepted vary significantly across jurisdictions. This is one of the key factors contributing to the divergent HTA procedures and outcomes observed in different countries, as previously discussed [29, 31, 43, 44]. Adding to the complexity, the development of innovative and personalized therapies is a fast-moving field that is inherently associated with new uncertainties, therefore heightening the challenges in clinical assessments and requiring new state-of-the-art types of methodologies and approaches to manage these [6, 45]. Hence, this emphasizes the importance of implementing guidelines as dynamic and living documents that would allow for continuous integration of new knowledge and novel methodologies to ensure that guidelines remain suitable not only at present, but also in the future. This action point pertains not only to uncertainty management but also holds a pivotal role in shaping clinical guidelines to ensure their relevance for JCAs and the PICO scoping process.

With regard to the data provided by the HTD, these may be considered insufficient for adequate assessment due to various reasons, inevitably resulting in evidence gaps that necessitate additional information [4, 46]. During the 2022 Spring Convention of the EAA, it already became evident that the recognition and resolution of these evidence gaps play a crucial role in tackling challenges associated with uncertainties in joint HTA [4]. Indeed, the first step is to define possible evidence gaps as early as possible (preferably during a JSC) while simultaneously preparing for post-launch data collection activities; however, participants of the present convention emphasized the importance of outlining a strategy with regard to the availability of this additional evidence. In this context, leveraging RWD to address evidence gaps is viewed as a significant opportunity under the Regulation [41, 46]. However, this also introduces novel complexities due to the quality issues associated with RWD and unequal acceptance of RWD in different jurisdictions [47, 48]. As ATMPs and oncology products, including these for rare cancers, will be first to assess under the EU HTA Regulation, there is an urgent need for new guidelines covering RWD and their integration into joint assessments and management of remaining uncertainties, including situations in which non-randomized controlled trial (RCT) data are acceptable for HTA assessment.

### Limitations and outlook

To reduce potential bias in the working group discussions, extensive efforts were made to create balanced WGs with a diverse representation of participants from different stakeholder groups, nationalities, personal/professional backgrounds and participation modes. Nevertheless, there was variation in participation across the break-out sessions, ranging from 14 to 18 participants per WG, while participation in the voting ranged between 12 and 18 participants. As a result, the distribution of participants may not have been entirely equitable among the groups and future research on a broader sample of participants might be warranted to confirm the results shown here. Additionally, while consistent approaches and reporting were sought in the four respective WGs by organizing pre-convention meetings, variations in the flow of the break-out sessions and differences in note-taking practices could have arisen due to varying leadership teams across the groups. Further, the ratings of “readiness” towards the Regulation were provided by distinct groups of participants among the four simultaneous break-out sessions. Therefore, direct comparisons between the sessions may be limited.

In summary, this study has identified and prioritized a number of action points that, according to multi-stakeholder participants of the EAA Convention, are essential for a successful implementation of the EU HTA R. An important upcoming task for the Commission as per the Implementation Rolling Plan [8] is to adopt Implementing and Delegated Acts to establish procedural rules that align with EU laws and ensure uniform conditions for their implementation [2]. Both general and detailed procedural rules for JSCs and JCAs, for example, will be adopted by means of Implementing Acts, a process in which EUnetHTA 21’s deliverables on methodological guidance will be taken into account [2]. As stipulated in the Rolling Plan, these Implementing and Delegated Acts are scheduled to be adopted between Q4 2023 and Q4 2024 [8]. Until then, uncertainties about current guidelines and methodologies of JCAs and JSCs persist, hampering MS’ and other stakeholders’ efforts to fully prepare and make their procedures ready for the joint work envisioned under the Regulation. Consequently, this may discourage MS from taking the lead in upcoming JCAs. Therefore, in addition to the discussed action points, the timely adoption of Implementing and Delegated Acts as well as maintaining transparent communication throughout this process will be crucial for the Regulation to be truly operational in January 2025.

## Conclusions

Implementation of an EU-wide HTA approach presents a unique opportunity for minimizing duplication of efforts and resources as well as ensuring the long-term sustainability of EU HTA cooperation, to ultimately achieve the goal of improving patient access to innovative health technologies. At “midterms” of the preparation phase towards the EU HTA Regulation, substantial challenges predominantly revolve around harmonization and standardization, capacity building and collaboration, uncertainty management and robust data provision. Moving forward in the remaining preparation phase, key priorities include tangible strategies to tackle those challenges and the timely adoption of Implementing and Delegated Acts, ultimately to establish an effective and fit-for-purpose process for common HTA starting in January 2025.

## Data Availability

The datasets used and/or analysed during the current study are available from the corresponding author upon reasonable request.

## Abbreviations

ATMP: Advanced therapy medicinal product
CG: Coordination Group
EAA: European Access Academy
EC: European Commission
EU HTA R: European Regulation on Health Technology Assessment
EU: European Union
EUnetHTA: European network for Health Technology Assessment
HTA: Health Technology Assessment
HTD: Health Technology Developer
IQR: Interquartile range
JCA: Joint Clinical Assessment
JSC: Joint Scientific Consultation
MS: Member States
PICO: Population, Intervention, Comparator(s), Outcomes
RCT: Randomized controlled trial
RWD: Real-world data
WG: Working group

## Declarations

The views expressed in this article are the personal views of the author(s) and should not be interpreted or cited as representing the stance of the regulatory agency/agencies or organization(s) with which the author(s) is/are affiliated.
